# Retaining Opportunities, Completing Key Projects with Remote Student Employees During COVID-19

**DOI:** 10.1177/1550190620980732

**Published:** 2021-06

**Authors:** Henry Handley, Kayla Harris

**Affiliations:** 1 University of Dayton Libraries, Dayton, OH, USA; 2 University of Dayton, Dayton, OH, USA

**Keywords:** COVID-19, collections management, case study, archives, subject focus, digitization, records, collections, library, staff and volunteers, cultural heritage, research and topics

## Abstract

As the field of higher education began furloughs and layoffs to alleviate COVID-19 budget concerns, cultural heritage workers were directed to clearly demonstrate how their work contributes to institutions’ educational missions. Although physical library and archival collections were deemed inaccessible and less critical during the pandemic than ebooks, electronic journals, and digitized special collections, the two special collections projects considered in this case study demonstrate the value of continuing collections management work remotely and the relevance of student employees and other contingent workers in libraries and archives. The projects—one an inventory and bibliography of books acquired from a defunct religious library, and the other a review of digitized audio cassette tapes with little content information outside of the audio itself—enabled the retention of student workers facing few summer job opportunities and ineligibility for unemployment insurance, providing additional experience as well as compensation during an economic, as well as public health, crisis.

Special collection libraries and archives contribute to the educational mission of academic institutions in many ways. Public-facing activities such as exhibits or class instruction are often the most well-known aspects of a special collection library at a university. However, these activities are often only possible because of the behind-the-scenes and ongoing collection management work that facilitated access to items. Likewise, student employees in libraries and archives often complete many of the overlooked, but essential day-to-day tasks necessary for access. From correctly shelving circulating materials to rehousing archives in acid-free boxes, these are the tasks completed by student employees that ensure collections are available for patrons now and in the future. In spite of being less visible than library and archives professionals, student employees are key to many special collections and their day-to-day functions. In the Spring 2020 semester, student employees’ remote work on two projects in the University of Dayton’s Marian Library spotlights the value and potential for collections management.

## COVID-19 in Context at the Marian Library

The Marian Library was founded in 1943 by the Society of Mary to advance the Marianist mission of making the Blessed Virgin Mary better known, loved, and served (University of Dayton 2020). The collections support scholarship and research of the International Marian Research Institute, the University of Dayton community, and researchers worldwide. These collections include 675 linear feet of archival materials, more than 12,800 pieces of artwork and artifacts, and more than 111,000 volumes of rare books, periodicals, and circulating books and media. Together these collections are a distinct expression of the university’s Catholic, and Marianist heritage. The department includes a Director, two librarians, one archivist, three professional staff employees, and a number of student employees depending on the semester.

On March 10, 2020, the University announced the suspension of in-person classes and closure of campus housing effective March 11. Marian Library employees started preparing projects that could be completed remotely by librarians, staff, and student employees in anticipation of a closure of other campus buildings. All University of Dayton (UD) employees were instructed to work remotely, role-permitting, starting the week of March 23, 2020, at which time the Marian Library, and the rest of the University Libraries closed to the public as well. Throughout the closure, Marian Library employees continued the essential functions of the library remotely, including research consultations, virtual class visits, and several specific projects related to collections management. Contactless circulating book pickup remained available by circulation staff. Electronic interlibrary loan services for journal articles and book chapters were also increased.

Non-essential spending was frozen as administrators reckoned with the early financial impacts of housing reimbursements and other immediate costs from the unfolding situation. On April 28, 2020, UD announced more than 500 staff furloughs and layoffs across campus as cost-saving measures in direct response to the impacts of the coronavirus and the uncertainty surrounding enrollment for Fall 2020 (Gnau 2020). Approximately 38% of Libraries staff were furloughed or laid off.^1^ The remaining library employees were instructed to prioritize the aspects of their roles that directly contributed to the educational mission of the institution. As a special collection it was important for the Marian Library to emphasize that collections management tasks, and the individuals who perform this work directly support the University’s educational mission by facilitating access to content.

## Collections Management Projects

**Figure 1.**
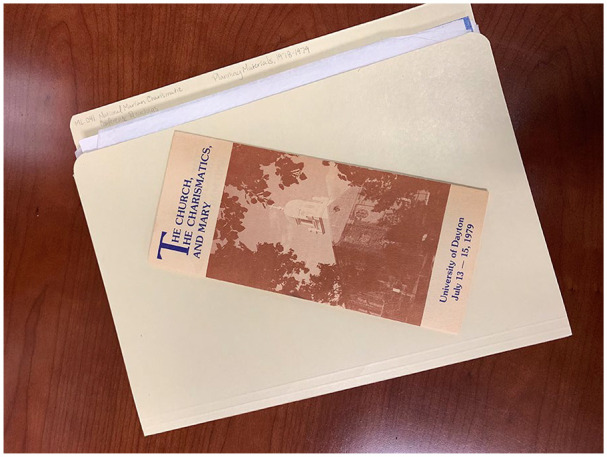
Brochure from the National Marian Charismatic Conference Collection, ML.041, highlighting the event held at the University of Dayton in 1979. *Source*. Courtesy of the authors.

Two collections management priorities were identified within the Marian Library: the digitization and eventual access to two audio cassette collections, and the processing of the Boeddeker book collection. The audio collections were digitized by SceneSavers in February 2020 as the first step towards making the content accessible. The Father Bertrand Clemens tapes from 1982 include lectures on several Marian topics, such as “Mary’s Humanness” while the National Marian Charismatic Conference tapes documented sessions from the National Marian Charismatic Conference hosted at the University of Dayton in both 1979 and 1980. This event featured several influential and well-known Catholic speakers, such as Catherine de Hueck Doherty, a social justice pioneer whose cause for canonization as a saint is under consideration by the Catholic Church (McNamara 2017). Although there are a few planning documents from the event in the collection such as a brochure (see Figure 1), the actual presentations do not exist in any other format. After digitization, additional metadata work still needed to be completed by the Marian Library before content could be made available through the University of Dayton’s institutional repository, eCommons.

The Boeddeker book collection was acquired by the Marian Library in April 2019 as a gift from the Franciscan School of Theology (FST) in San Diego, California. Father Alfred Boeddeker, O.F.M. acquired the approximately 1,500 books for the Marian Center and Library while serving as a priest at St. Boniface Church in San Francisco, California. Fr. Boeddeker was a prominent 20th century Marian theologian and leader in Mariology who was also noted for founding St. Anthony’s Dining Room and other health, education, and housing programs for people living in poverty in the Bay Area (Associated Press 1994). The books represent both his reading experience and the Marian books he selected for library patrons.

**Figure 2.**
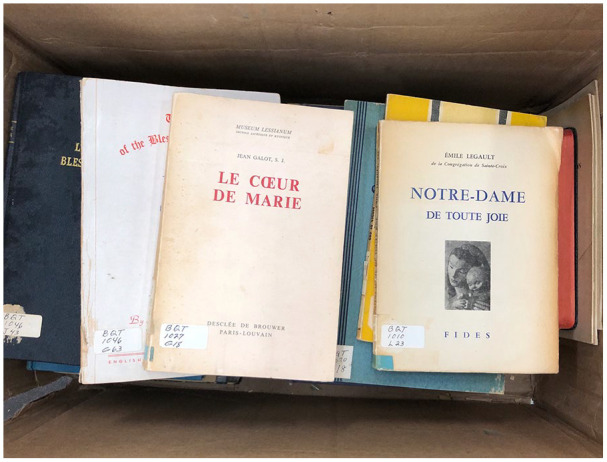
Books from the Marian Library’s Boeddeker collection, as they arrived in 2019. *Source*. Courtesy of the authors.

The acquisition was a years-long process: a deed of gift was signed in July 2016, but the books remained in poor storage conditions in California and at one point even went missing. As student employees inventoried 671 unique titles in the Boeddeker collection (and numerous duplicate copies) from August 2019 to March 2020, they identified dust, damaged bindings, stains, and mildew as Figure 2 illustrates. Thirteen boxes were determined to be too moldy to be safely reviewed and were deaccessioned. In light of the collection’s overall condition and the large number of duplicates, the Marian Library collections librarian and director decided that the main goals of processing the collection were creating an inventory and bibliography of the books for researchers; preserving and cataloging books not already in the Marian Library’s collection; and, not least, protecting the health of library workers handling the books.

## Student Employees and the Libraries

Against this backdrop, student employees in the Marian Library were assuming increasing responsibility for independent work in collections management. Overall, the University of Dayton Libraries were pursuing experiential learning opportunities for student employees in many positions, in particular in frameworks that editors Sara Arnold-Garza and Carissa Tomlinson identified in Students Lead the Library: students and student groups serve as library employees, curators, ambassadors, consultants, leaders, and designers. In 2019 to 2020, initiatives included a mini-grant awarded to further vocational discernment among University Libraries student employees; blog posts by student employees and interns; and professional development programming on resume-building, interview etiquette, and more.

Marian Library student employees assist patrons, page books, shelve, shelf-read, and scan materials. As special collections workers, Marian Library student employees also complete collection management tasks, such as review various areas of the collections, including microfilm, journal holdings, and article file materials.

At the beginning of the Spring 2020 semester, 11 student employees were working for the Marian Library. Four student employees worked in back-of-house roles, four in front-of-house (primarily at the front desk, although there was some crossover), two in the Marian Library’s art collection across campus in Fitz Hall, and one in archives.

Student supervisors sought to hire and retain students who supported essential, and more complex, collection management work with their skills and experience (as demonstrated in, for example, previous work in data entry, research assistance, and libraries). Four people were official or de facto supervisors: the Marian Library administrative assistant served as the supervisor and coordinator for all students and oversaw hiring, time reporting, and front desk projects, and three supervisors directed student work in archives, published materials, and art respectively.

## Students Continue Mission-Critical Projects Remotely

After students left campus on March 11, several Marian Library students requested to continue their positions remotely. With the many different priorities on campus during the transition to remote employee work and online instruction, the university administration did not initially provide clear guidance on whether this was a possibility for students on-campus jobs. Absent university directives, the Marian Library director and dean of the University Libraries initially approved remote work for student employees.

Career Services offered student employees receiving federal work study financial aid a choice between being paid an average of their weekly earnings or continuing to work if their core job duties could be performed remotely. A second message the next day on the university’s internal website, Porches, announced that all student employees across the university could continue working remotely, but again only if they could meet core responsibilities. Five Marian Library student employees who had requested to continue working were now permitted to do so.

With this initial uncertainty in mind, Marian Library student supervisors selected a variety of remote projects for students, including some short-term initiatives that could be more easily postponed if necessary. The Boeddeker bibliography made it possible to continue the inventory project. It contains essential publication information from the inventory and is intended to be expanded once the inventory can be completed in person. Student employees also noted books from the inventory that need further, in-person investigation; this will include reviewing titles present in several editions and/or from several publishers. The time-sensitive preservation issues in the Boeddeker collection mean that students’ significant progress on the project is essential to the collection’s ongoing preservation in the Marian Library.

In developing the remote work project for the archival student employee, a crowdsourced document started by Lydia Tang and the Society of American Archivists Accessibility and Disability Section (ADS), proved a useful resource for potential ideas. As Tang explained in an interview with the Council on Library and Information Resources (CLIR), “We wanted this document to be an advocacy tool for archivists to show administrators how they can still do impactful work, even without physical access to their collections.” (Tang 2020). The digitization of the cassette tapes completed by SceneSavers only accomplished part of the project’s overall goal to make the content accessible. Both collections contained duplicates and shortly after SceneSavers received the tapes, their project manager explained that all tapes would be digitized. The labels did not identify master copies, and each tape seemed to have varying levels of sound quality. It would be up to Marian Library personnel to select which files had the best sound quality and completeness to use as access files for patrons.

The archives student employee had been employed in the Marian Library for over two years and had worked in many capacities, including physical processing of collections, assistance with exhibits and class visits, and detailed data clean-up in the archival content management system, ArchivesSpace. When the audio files were returned to the Marian Library, the student completed a quality check of the contents on the hard drive. With the shift to remote work, it was a departmental priority to continue progressing on collection management projects, especially those already underway, and retain expertise of trained student employees. Building off the initial quality control check, the archives student created a tracking spreadsheet that noted issues of quality such as volume, hissing or clicks, background noise, and length of recording. The spreadsheet also noted subject keywords for future access points when the files are integrated in the content management system. This thorough inventory of the digital files identified duplicate recordings, and recommended a best version in the case of multiples. While the overall project is still in process, documenting these issues and identifying the best versions is the next step in the process towards making these files accessible to users.

As students finished the Spring 2020 semester online, they faced continuing uncertainty about summer jobs, internships, and whether they would be able to safely return to campus in the fall. Marian Library student employees were notified in April that summer hours for either remote or on-campus work would not be available. Friday, May 1 marked student employees’ last day working remotely and the first day of furloughs for University Libraries staff, including those in the Marian Library.

## Advocating for Student Employees

For the Fall 2020 semester, the university has planned for a residential campus experience that calls for certain positions (including student employees in the Libraries) to work on campus. Based on increased COVID-19 infection rates in Ohio and in Dayton during the summer of 2020 and the uncertainty regarding in-person instruction and research in higher education, the authors do not know whether student employees can continue to work on campus throughout the fall. Yet, the success of these projects are useful tools in advocating for their positions and their professional development in them, in whatever form that may take.

Student workers’ experience and ability to complete critical collection management work helped justify their continued spring semester employment after campus closure. This demonstrated that collection management is not limited to physical materials, and can continue as remote work for libraries and archives employees at all levels. As Tang explains, “this COVID-19 shutdown hopefully has helped clarify that archives are more than physical records in buildings and that archival labor doesn’t have to be in an office.” This is likewise true for all cultural heritage collections. As the Information Maintainers Collective notes, “times of reassessment can be pivotal to the justification of information maintenance” (The Information Maintainers et al. 2019, 11) and thus collection management is maintenance. The retention of student employees during the spring semester kept a focus on this maintenance. In addition, all student employees who did not graduate in May 2020 have returned to continue working with the Marian Library in Fall 2020. One student has also volunteered for the University Libraries Committee. Another student reflected that “being able to continue working remotely was something to look forward to with everything else that was going on. It felt like I could continue to make meaningful contributions.” This student also explained how valuable it was to continue working at UD where work shifts were scheduled around classes, as opposed to having to seek other employment at an already stressful time. The work of student employees highlights the essential nature of the ongoing, long-term collections management necessary for two of the Marian Library’s hidden collections.

## Footnotes

1. Citing privacy, the university did not share the specific number of furloughs and layoffs in each unit, including the University Libraries. The authors referred to a temporary all-staff mailing list that did not include furloughed and laid-off staff.
